# Impact of previous treatment history and B-cell depletion treatment duration on infection risk in relapsing-remitting multiple sclerosis: a nationwide cohort study

**DOI:** 10.1136/jnnp-2023-333206

**Published:** 2024-05-14

**Authors:** Suvi Virtanen, Fredrik Piehl, Thomas Frisell

**Affiliations:** 1 Department of Medicine Solna, Karolinska Institutet, Stockholm, Sweden; 2 Department of Clinical Neuroscience, Karolinska Institutet, Stockholm, Sweden

**Keywords:** MULTIPLE SCLEROSIS

## Abstract

**Background:**

B-cell depletion displays striking effectiveness in relapsing-remitting multiple sclerosis (RRMS), but is also associated with increased infection risk. To what degree previous treatment history, disease-modifying therapy (DMT) switching pattern and time on treatment modulate this risk is unknown. The objective here was to evaluate previous DMT use and treatment duration as predictors of infection risk with B-cell depletion.

**Methods:**

We conducted a nationwide RRMS cohort study leveraging data from the Swedish MS registry and national demographic and health registries recording all outpatient-treated and inpatient-treated infections and antibiotics prescriptions from 1 January 2012 to 30 June 2021. The risk of infection during treatment was compared by DMT, treatment duration, number and type of prior treatment and adjusted for a number of covariates.

**Results:**

Among 4694 patients with RRMS on B-cell depletion (rituximab), 6049 on other DMTs and 20 308 age-sex matched population controls, we found higher incidence rates of inpatient-treated infections with DMTs other than rituximab used in first line (10.4; 95% CI 8.1 to 12.9, per 1000 person-years), being further increased with rituximab (22.7; 95% CI 18.5 to 27.5), compared with population controls (6.6; 95% CI 6.0 to 7.2). Similar patterns were seen for outpatient infections and antibiotics prescriptions. Infection rates on rituximab did not vary between first versus later line treatment, type of DMT before switch or exposure time.

**Conclusion:**

These findings underscore an important safety concern with B-cell depletion in RRMS, being evident also in individuals with shorter disease duration and no previous DMT exposure, in turn motivating the application of risk mitigation strategies.

**WHAT IS ALREADY KNOWN ON THIS TOPIC**

**WHAT THIS STUDY ADDS**

- Following a nationwide relapsing-remitting multiple sclerosis cohort exposed to different therapies over 9 years, we found a doubled rate of serious infections on rituximab compared with non-B cell-depleting therapies combined. The increased risk of infection with rituximab was not modulated by previous multiple sclerosis therapies or the duration of treatment.

**HOW THIS STUDY MIGHT AFFECT RESEARCH, PRACTICE OR POLICY**

- Infection risk mitigation strategies are motivated with B-cell depletion in RRMS regardless of treatment history and duration of exposure to B-cell depletion.

## Introduction

 The treatment landscape of multiple sclerosis (MS) has evolved drastically over the last decade, with the introduction of increasingly effective disease-modifying therapies (DMT).^1 2^ While newer treatment options offer stronger suppression of MS inflammatory disease activity compared with older DMTs, they may also be associated with particular treatment-related risks.

Infections, in particular, constitute a primary concern in the era of modern MS treatments,^3 4^ with evidence for differences in qualitative and quantitative aspects of infection risk depending on the mode of action of DMTs. For example, natalizumab is known to increase the risk of JC virus reactivation and progressive multifocal leukoencephalopathy, while fingolimod has been associated with an increased risk of herpes reactivation.^5^,^7^ Depletion of B-cells represents an increasingly used treatment modality in both relapsing-remitting MS (RRMS) and progressive MS, and has been associated with primarily bacterial infections compared with platform therapies in observational settings.^7^ Interestingly, though B-cells play an important role in physiological immune functions, neither the rate of mild nor severe infections differed significantly compared with control treatment arms in the registration studies for ocrelizumab and ofatumumab.^8 9^ However, observation time in these trials was limited, and it has been suggested that infection risk increases with longer exposure to B-cell-depleting therapies.^10^,^12^ In line with this, laboratory parameters associated with increased infection risk, primarily hypogammaglobulinemia, may develop with longer treatment exposure, and lack of B-cells is associated with reduced humoral vaccination responses.^13 14^ Emerging data also indicates that infection risk may be greater in people with MS (PwMS) treated in routine clinics compared with clinical trial populations.^10^ This also includes an increased risk of severe COVID-19 with B-cell depletion compared with other treatment modalities.^15 16^

We previously reported an increased risk for hospital-treated infection with rituximab, a B-cell depleting drug used off-label for MS, compared with older platform self-injected MS DMTs in a nationwide cohort study.^7^ Leveraging a larger study cohort with longer follow-up time, we here sought to refine risk estimates and, in particular, to what degree time on treatment and treatment history impacted infection risk with B-cell depletion. The specific objectives of the study were to address if prior exposure to MS therapies predicts the risk of infection during treatment with rituximab, and secondarily, if infection risk is modified by treatment history when a DMT switch is performed from another DMT to rituximab or by longer exposure to treatment.

## Methods

We performed a nationwide cohort study by linking data on MS treatment and patient characteristics in the Swedish MS Register (SMSreg) to national health registries. The SMSreg is used in all Swedish neurology departments and has an estimated national coverage of about 80% of prevalent MS cases with high validity of registered data.^17 18^ The national health registries have virtually complete coverage, and for this study comprised the Total Population Register with information on demographic variables, the Swedish Patient Register with information on inpatient (since 1964 and with national coverage since 1987) and outpatient (since 2001) specialised care diagnoses coded according to Swedish revisions of the International Classification of Diseases codes, the Causes of Death Register (since 1952) and the Swedish Prescribed Drug Register with information on all collected prescribed drugs (since 2005).

### Study cohort

The primary study cohort comprised all PwMS (age ≥18) with RRMS in SMSreg who started rituximab for the first time from 1 January 2012 to 30 June 2021 (N=5999). Incidence rates (IR) of infection in the main cohort were compared with rates among PwMS who, in the same period of time, initiated any of: injectables (N=2250; comprising interferons and glatiramer acetate), natalizumab (N=1990), fingolimod (N=1552) or dimethyl fumarate (N=2745). Since the aim was to estimate risks attributable to current therapy, follow-up was censored at treatment discontinuation (ie, ‘on drug’-analyses). To further benchmark infection rates, we created a population-based comparator group of MS-free individuals by matching rituximab initiators 1:5 to subjects randomly drawn from the Swedish population (N=22 806). To avoid immortal time bias due to possible retrospective recording of data in the SMSreg, patients with MS with the start of index treatment >90 days before recorded inclusion in SMSreg were excluded (N=1492). Individuals with any record of infection within 180 days before the index date were excluded to avoid ongoing infections (PwMS=2117, general population comparators=2498). Patients whose treatment history included mitoxantrone or stem cell transplantation were also excluded (N=56), as were those who started injectables, glatiramer acetate, natalizumab, fingolimod or dimethyl fumarate after treatment with rituximab (N=128).

### Outcomes

The main outcome was time to first severe infection, defined as hospitalised infection or death due to infection. The two secondary outcomes were; (1) time to specialist care outpatient infections, and (2) time to any filled prescription of a systemic antibiotic. Details on the outcome definition are available in the online supplemental table S1.

### Exposures and follow-up time

Time at risk was counted from the treatment start date (index date) until the outcome of interest, 90 days after recorded discontinuation of therapy (recorded stop of therapy or start of a new therapy, if earlier), emigration, death, exclusion from SMSreg, start of mitoxantrone or haematogenic stem cell therapy, or data extraction date (30 June 2021), whichever occurred first. For the general population comparator group, the index date was defined by their matched patient’s rituximab initiation date, and follow-up ended if a diagnosis of MS was made. An individual who started more than one DMT during the observation period could contribute data to multiple treatment periods.

To assess if infection rates varied by first versus later treatment line, and to put infection rates on rituximab in the context of other DMTs and the general Swedish population, we first divided the full cohort into five exposure groups: (1) treatment naïve PwMS starting rituximab (rituximab first line), (2) PwMS switching to rituximab from another therapy (rituximab later line), (3) treatment naïve PwMS starting injectables, natalizumab, fingolimod or dimethyl fumarate (other DMT first line), (4) PwMS switching to injectables, natalizumab, fingolimod or dimethyl fumarate from another non-rituximab DMT (other DMT later line) and (5) population-based MS-free controls.

To further investigate the impact of previous DMT, we then restricted the sample to PwMS switching to rituximab from another therapy within 180 days, stratified on type of DMT switching from; (1) natalizumab, (2) injectables, (3) dimethyl fumarate and (4) fingolimod.

Finally, to assess the impact of the duration of rituximab treatment, we included all subjects starting rituximab and split the follow-up time by year since treatment start.

### Covariates

Other covariates and potential confounders were assessed at the index date. Demographic covariates included age, sex, region of residence, country of birth (categorised as Sweden or other) and highest educational level (categorised as ≤12 years or >12 years). Medical history was assessed from any filled prescription or diagnosis in the 5 years before treatment started and included any serious inpatient treated infection, outpatient infection treated in specialist care, systemic antibiotics and a modified Charlson Comorbidity Index.^19^ MS-specific covariates included time since diagnosis, any relapse within 1 year prior to treatment start, Expanded Disability Status Scale (EDSS) score and Multiple Sclerosis Impact Scale (MSIS-29) physical and psychological score. The number of previous treatments was included as a covariate in the analysis of the rituximab switch group. The year of treatment start was included to account for any differences in the risk for infection or recorded treatment according to the calendar year, and the general population comparator group was assigned a start year based on the date of start of index treatment for the matched patient with MS.

### Statistical analyses

Tabulations for each exposure group were made for baseline patient characteristics, and number of events. The cumulative incidence of serious infections by time on rituximab was plotted as one minus the Kaplan-Meier survival estimate, and hazards plotted by Epanechnikov Kernel-smoothing of Nelson-Aalen estimated cumulative hazard increments as implemented in the *muhaz* package in R. IR per 1000 person-years with 95% CIs were calculated for all outcomes and Cox proportional hazard models were fitted to estimate HR for occurrence of first ever infection after treatment start, with time since treatment start date as the time scale in analyses of DMT history, and age as time scale in analysis of duration of rituximab use. To assess the impact of different covariates, a series of models were run, incrementally adding domains of the covariates listed earlier. Age was modelled with second-degree polynomials. Robust SEs were used to account for observations repeatedly included for multiple treatment starts. There was no missing data in most variables derived from national registers, but EDSS and MSIS-29 were missing for 49.9% and 34.3% at index date. The latter two were therefore categorised into quartiles, with an indicator for missing data included in the analyses. Cumulative incidence and hazard were plotted using R V.4.3.1, while SAS V.9.4 was used for all other statistical analyses.

### Sensitivity analyses

We performed two sensitivity analyses. First, to exclude potential bias introduced by the COVID-19 pandemic, all analyses were run with follow-up ending 29 February 2020. Second, the analysis of the impact of previous DMTs was run restricted to participants who had been treated at least for 1 year with the previous DMT.

## Results

In total, we included 10 743 PwMS and 20 308 population-based controls in the full cohort, with 1458 participants in the first-line rituximab group and 3236 in the later line group, and 2774 and 3275 in the corresponding other MS DMT groups. Baseline characteristics are detailed in table 1. In brief, the two first-line groups had a shorter disease duration, a higher proportion with a history of recent relapse and lower mean age at the start of index therapy compared with the later-line groups. Comorbidity burden, disability status (EDSS) and self-reported impact of MS (MSIS-29) were similar both by line of treatment and DMT. Proportions with a history of inpatient-treated infection and use of systemic antibiotics prior to DMT start were greater in the later-line compared with first-line cohorts, while the history of outpatient-treated infections was similar. The year of start of index therapy (or matched year for the MS-free population) varied between groups, with earlier start in the other DMT later line group and the most recent start year in the first-line rituximab group. Exposure groups also differed regarding area of residence, with increased proportions of individuals exposed to rituximab in the regions of Northern Sweden and Stockholm, compared with the rest of Sweden.

**Table 1:** Baseline characteristics in the full cohort

	Rituximab first line	Rituximab later line	Other DMT first line	Other DMT later line	General population
Number of observations	1458	3236	2774	3275	20 308
Start year, median (IQR)	2018 (2016–2019)	2017 (2015–2018)	2015 (2014–2018)	2014 (2013–2016)	2017 (2016–2019)
Age in years, mean (SD)	38.0 (11.3)	40.4 (10.4)	36.2 (10.6)	39.3 (10.3)	39.6 (10.7)
Female, n (%)	937 (64.3)	2282 (70.5)	1904 (68.6)	2341 (71.5)	13 683 (67.4)
Born in Sweden, n (%)	1231 (84.5)	2769 (85.6)	2328 (84.0)	2847 (87.0)	16 373 (80.6)
Education, 12+ years, n (%)	669 (46.6)	1490 (46.4)	1227 (45.0)	1553 (47.8)	9342 (46.7)
Years since MS diagnosis, mean (SD)	1.0 (3.4)	7.6 (6.0)	1.0 (3.4)	6.5 (5.6)	
Hospitalised infection 5 years before DMT, n (%)	36 (2.5)	126 (3.9)	79 (2.8)	107 (3.3)	501 (2.5)
Infection outpatient care 5 years before DMT, n (%)	208 (14.3)	475 (14.7)	355 (12.8)	445 (13.6)	2224 (11.0)
Antibiotics 5 years before DMT, n (%)	737 (50.5)	1893 (58.5)	1461 (52.7)	1945 (59.4)	9832 (48.4)
Charlson Comorbidity Index, mean (SD)	0.2 (0.6)	0.1 (0.6)	0.2 (0.6)	0.1 (0.5)	0.1 (0.5)
Any relapse year before DMT, n (%)	517 (35.5)	648 (20.0)	1114 (40.2)	860 (26.3)	
EDSS, mean (SD)	1.7 (1.3)	1.8 (1.4)	1.5 (1.3)	1.7 (1.4)	
MSIS-29 physical, mean (SD)	1.8 (0.8)	1.8 (0.8)	1.8 (0.8)	1.8 (0.8)	
MSIS-29 psychological, mean (SD)	2.4 (1.0)	2.2 (0.9)	2.4 (1.0)	2.2 (0.9)	
Duration of previous DMT, years, mean (SD)		3.4 (3.4)		3.4 (3.6)	
History of natalizumab, n (%)	0 (0.0)	1196 (37.0)	0 (0.0)	480 (14.7)	
History of fingolimod, n (%)	0 (0.0)	560 (17.3)	0 (0.0)	238 (7.3)	
History of dimethyl fumarate, n (%)	0 (0.0)	847 (26.2)	0 (0.0)	354 (10.8)	
No previous DMT, n (%)	1458 (100.0)		2774 (100.0)		
1 previous DMT, n (%)		1560 (48.2)		2188 (66.8)	
2 previous DMTs, n (%)		1083 (33.5)		836 (25.5)	
3+ previous DMTs, n (%)		593 (18.3)		251 (7.7)	
Reason for previous treatment stop, n (%)					
Adverse events		719 (22.3)		974 (29.8)	
Loss of effect		1117 (34.6)		1329 (40.7)	
Other		1390 (43.1)		963 (29.5)	

DMTdisease-modifying therapyEDSSExpanded Disability Status ScaleMSmultiple sclerosisMSIS-29Multiple Sclerosis Impact Scale

### Infection risk on rituximab versus other DMTs

**Figure 1:**
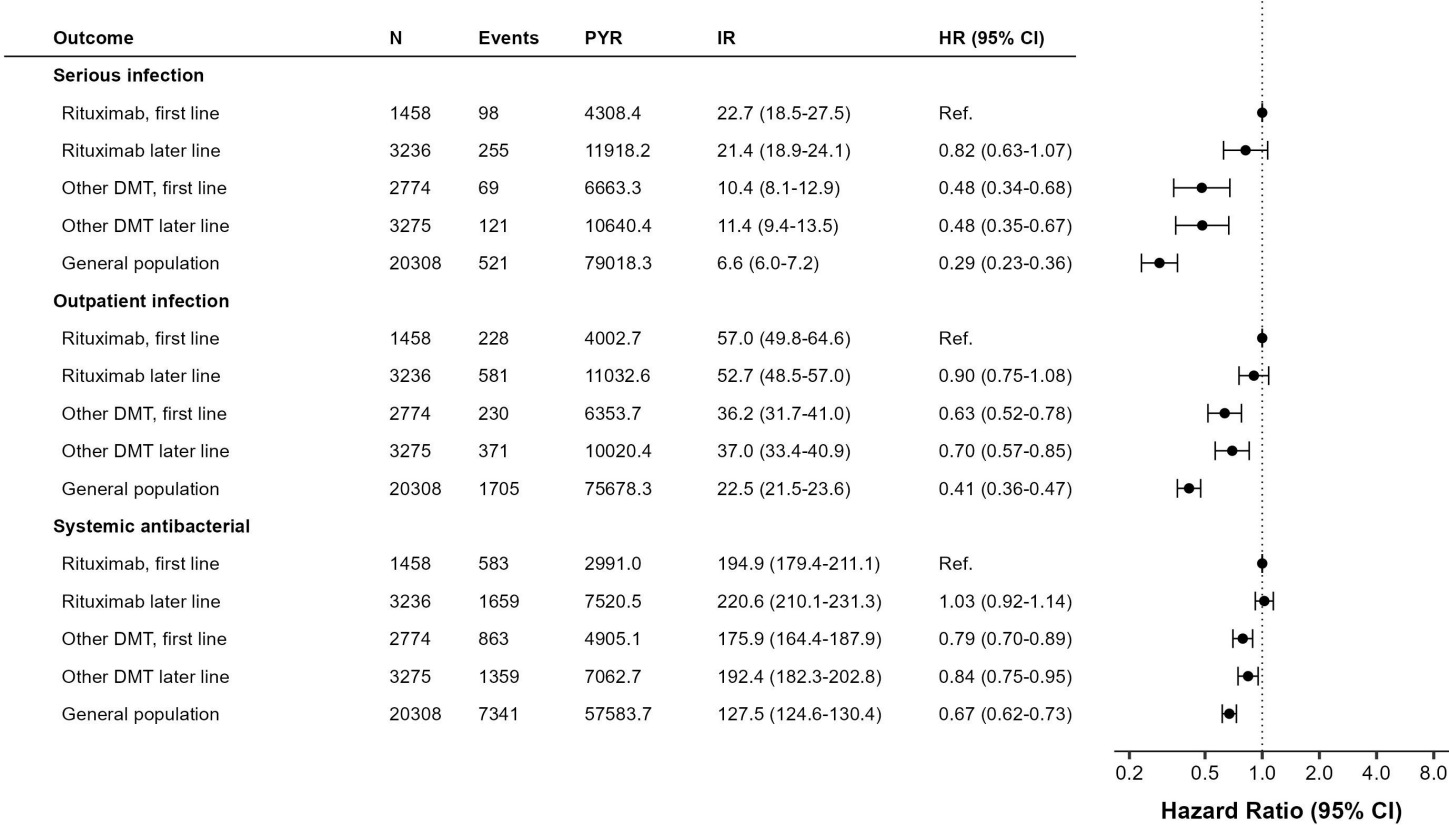
Infection rate in rituximab as first or later line therapy compared with alternative DMTs and the general population. DMT, disease-modifying therapy; events, number of patients with infection; HR, HR with 95% CI from Cox proportional hazards model adjusting for age, sex, start year, comorbidities, demographics and (comparison between MS cohorts only) MS clinical characteristics; IR, incidence rate per 1000 PYR; N, number of patients; PYR, person-years of follow-up.

Serious (inpatient treated or fatal) infections were more frequent in the two rituximab cohorts compared with the corresponding other DMT groups, and did not differ between first or later line use (figure 1). Thus, IRs for serious infection were 22.7 versus 21.4 events per 1000 person-years (PYR) in the first and later line rituximab groups, respectively. This was not meaningfully impacted by adjustment for measured confounders (HR from the most adjusted model was 0.82, 95% CI 0.63 to 1.07, for intermediate models see online supplemental table 2). Corresponding rates with other DMTs were significantly lower, 10.4 and 11.4 in the first and later line cohorts, respectively, and 6.6 among MS-free controls. Adjusted HRs for serious infections from Cox regression estimated a roughly halved risk with other DMTs compared with rituximab, and reduced by two-thirds in the MS-free population. A slightly reduced magnitude of relative risk across groups were recorded for outpatient treated infections but the pattern remained similar; the first-line rituximab group displayed the highest IR with 57.0 events per 1000 PYR, followed by 52.7 in the rituximab-later line group, 36.2 and 37.0 in the other DMT first and later line group, respectively, and 22.5 in the control group. In contrast, IR of prescription of systemic antibiotics was numerically highest in the rituximab later-line group, though not significantly higher than with rituximab first line, and with a pattern otherwise remaining similar to previous outcomes. Three cases of progressive multifocal leukoencephalopathy were recorded during follow-up; two in natalizumab-treated patients and one in the rituximab later-line group, diagnosed soon after the switch from natalizumab. Nine fatal infections were recorded, six in the control group and three among PwMS, all of whom were or previously had been treated with rituximab.

### Infection risk on rituximab by previous DMT

**Figure 2:**
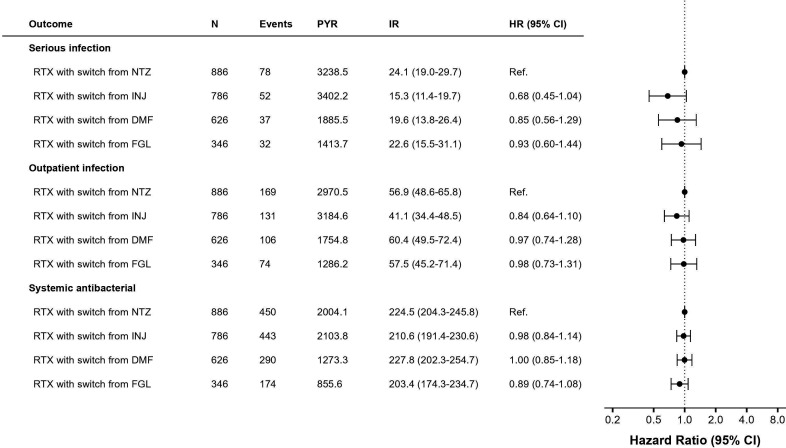
Infection rate in rituximab switching from natalizumab compared with switches from injectables, dimethyl fumarate and fingolimod. DMF, dimethyl fumarate; events, number of patients with infection; FGL, fingolimod; HR, HR with 95% CI from Cox proportional hazards model adjusting for age, sex, start year, comorbidities, demographics, MS clinical characteristics and treatment history; INJ, injectables; IR, incidence rate per 1000 PYR; N, number of patients; NTZ, natalizumab, PYR, person-years of follow-up; RTX, rituximab.

Next, we explored the risk of infection with a switch to rituximab from specific DMTs (figure 2). This restricted cohort included 2644 PwMS switching to rituximab from another DMT, with 886 switching from natalizumab, 786 from injectables, 626 from dimethyl fumarate and 346 from fingolimod, respectively (descriptive statistics in online supplemental table 3). IRs for serious infections ranged from 15.3 events per 1000 PYR with the switch from injectables, to 24.1 with the switch from natalizumab, though with overlapping 95% CI (figure 2). A similar non-significant trend for lower risk with the switch from injectables was observed also for outpatient infections, but not with the prescription of antibiotics.

### Infection risk by time on rituximab

**Figure 3:**
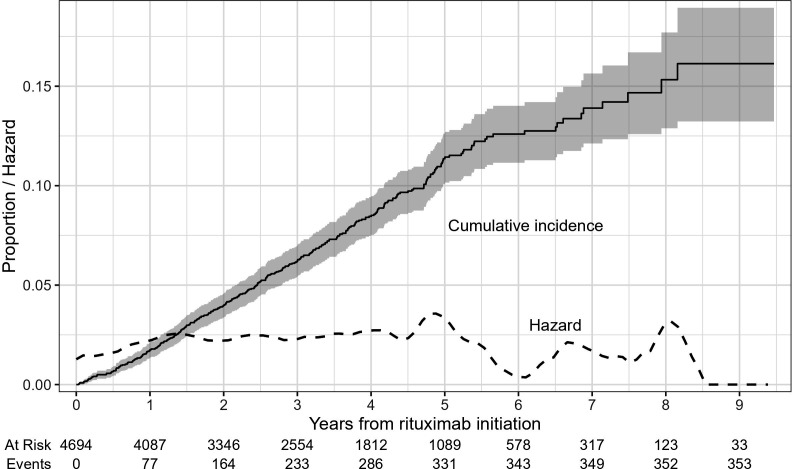
Cumulative incidence (95% CI) and hazard of serious infection while on rituximab.

**Figure 4:**
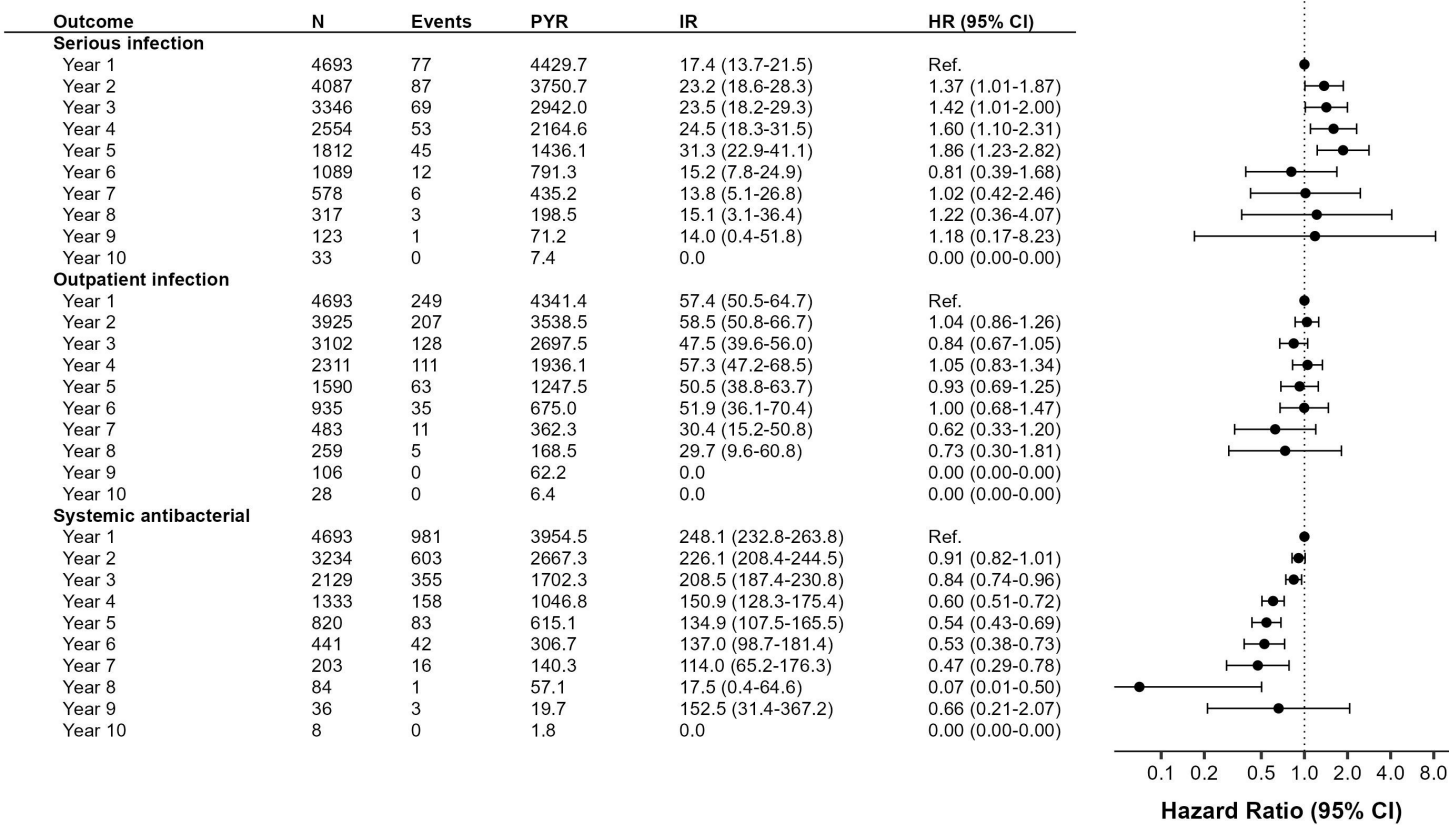
Infection rate in rituximab by time on treatment. events, number of patients with infection; IR, incidence rate per 1000 PYR; N, number of patients; PYR, person-years of follow-up; HR, HR with 95% CI from Cox proportional hazards model adjusting for age, sex, start year, comorbidities, demographics and MS clinical characteristics.

To address whether the duration of exposure to rituximab impacted infection risk, we plotted the Kaplan-Meier estimate of the cumulative incidence and Kernel-smoothed hazard curves for the first occurrence of serious infections (figure 3). As expected the proportion of patient who experienced at least one serious infection increased over time, but there was no indication that the hazard of experiencing an infection increased over time. The population remaining on rituximab treatment may have changed in meaningful ways over time, as the people discontinuing treatment may differ from those who remain. The drug survival has previously been shown to be higher on rituximab than on other DMTs; in this material 90% remained on rituximab 3 years after treatment initiation, with about 60% remaining on rituximab after 8–9 years (the latter with high uncertainty given that only 37 patients remained at 9 years) (online supplemental figure S1). Testing the difference in hazards over time in multivariable Cox regression we found that accounting for differences in baseline covariates between patients with long versus short treatment duration did not have a strong impact on the observed association with treatment duration. The hazard was significantly increased in years 2–5 compared with the first year of exposure, but with no significant risk increase thereafter (figure 4). Considering both the shape of the hazard curve and the pattern of adjusted HRs, infection risk appeared lower in the first exposure year, stable or marginally increasing in years 2–5, and then unchanged. The estimate for year 5 looks a bit higher, but any trend this may have indicated was not confirmed by later years, and the possible trend was further attenuated in the sensitivity analysis ending follow-up at the arrival of the COVID-19 pandemic (see next section). Lack of support for an increased risk with longer exposures to rituximab was more evident with outpatient infections and antibiotics use, the latter even showing a significantly lower hazard over time (figure 4).

### Sensitivity analyses

Finally, we performed two sets of sensitivity analyses. In the first, we censored follow-up on 29 February 2020 to exclude a possible bias introduced by the COVID-19 pandemic. This reduced somewhat the IRs for serious infections, while rates of antibiotics prescriptions increased, but differences between rituximab and the other DMTs remained similar (online supplemental table 4). Findings were similar also in the analysis on the rituximab switch cohort, with a tendency for lower point estimates for serious infections and higher for antibiotics use (online supplemental table 5). No differences in the incidence rate of serious or outpatient treated infections, by the duration of rituximab exposure, were statistically significant after censoring follow-up during the COVID-19 pandemic (online supplemental figure 2).

In the second sensitivity analysis, we restricted the study population of the rituximab switch cohort to those with ≥1 year of exposure to the previous therapy, but with no major impact on the results (online supplemental table 6).

## Discussion

In this large cohort study leveraging high-quality nationwide population-based data, we corroborate and extend prior observations on infection risks across different MS DMTs, with special reference to B-cell depletion. The cohort partly overlaps with our previous study,^7^ but with a larger RRMS cohort (10 743 vs 8519) and longer observation time (until June 2021 vs December 2017), thereby generating more precise risk estimates. In the prior study, we observed HRs of serious infections with non-B-cell depleting DMTs in the range of 0.59 to 0.77 compared with rituximab, against 0.48 in this data set, thus, indicating a higher risk increase. Most importantly, however, we here also addressed two potentially important covariates, namely the impact of prior treatment history and rituximab exposure time. Contrary to our expectations, we did not detect any meaningful differences between treatment-naïve and previously-treated PwMS, neither at the group level, nor with specific prior DMTs, although there was a trend for lower risk with a switch to rituximab from injectables than highly effective DMTs.

We also did not detect a clear trend for increased infection risk with longer exposure to B-cell depletion, except that the risk was lower in the first year of treatment compared with later on. This observation appears to contrast with a recent Norwegian study concluding that treatment duration with rituximab was a predictor for risk of hospitalisation for infection.^11^ Similarly, observational studies with different B-cell depleting drugs (rituximab, ocrelizumab, ofatumumab) in mixed patient populations have indicated an increased risk of development of hypogammaglobulinemia and infectious events over time.^10^ ^12^ ^20^ Apart from being based on smaller cohorts (n=184, 291, 447 and 565, respectively) and with more heterogenous case mixes, two of the studies compared patients with versus without infections at any point during follow-up,^10 12^ and one compared the annualised infection rate in the first two treatment years with treatment years three and beyond while only including patients who had been treated with B-cell depletion for at least 2 years thus requiring drug survival in the first but not in the second period of comparison.^20^ Besides the substantially larger study population, enabling more precise risk estimates, we here also controlled for a much wider range of confounders, although this only modestly impacted results and therefore remains an unlikely explanation for these discrepant results. Instead, we note that analysing infection as a binary end-point without accounting for the time scale corresponds to an analysis of the cumulative incidence, which would inherently be expected to increase with follow-up time (*cf*. our figure 3). In line with this interpretation, the Norwegian study analysed the presence of infection as a binary event (in a total of 57 subjects with infection) and found an adjusted OR of infection per treatment year of 1.52 (95% CI 1.11 to 2.09), but simultaneously noted that there was no evidence for an increase in IR over treatment year.^11^ The difference between studies may thus be an artefact of different handling of timescales. We cannot exclude, however, that differences in clinical management may also play a role. The Swedish MS society recommends a low-dose rituximab protocol with 500 mg of rituximab as a single infusion every 6 months, and that dose interval extension should be considered with the occurrence of hypogammaglobulinemia or infections.

It also deserves to be noted that the risk of hypogammaglobulinemia may differ depending on the type of drug and doses used. We previously conducted a real-world study comparing the impact of immunoglobulin G (IgG) concentrations with the Swedish low-dose rituximab protocol compared with standard dosing of ocrelizumab, indicating no significant drop over the first year with the former, but a mean 0.16 g/L drop with each infusion with the latter.^21^ Similarly, a systematic review of studies with ofatumumab and ocrelizumab in MS suggests that the impact on IgG concentrations is lesser with ofatumab.^22^ Therefore, we cannot exclude that an increased infection risk over time could become evident with the use of standard dosing at regular intervals of ocrelizumab or higher doses of rituximab due to greater proportions of developing hypogammaglobulinemia.

Interestingly, recent observational data suggest that the risk of recurrence of disease activity is low even with substantially longer treatment intervals introduced during the COVID-19 pandemic.^23 24^ Additionally, compared with regular intervals, extended dosing intervals with repopulation of B cell are associated with restoration of humoral responses to viral infections and vaccinations.^25^,^27^ Although it seems biologically plausible that treatment with anti-CD20 therapy over extended time periods should increase infection risks, our data instead suggest that in clinical practice the risk increase over time is limited. Although not tested in this study, it may therefore be speculated if current risk-mitigation strategies have blunted an increased risk over time that otherwise would have been seen.

In this study, we also analysed milder outpatient-treated infection and prescription of systemic antibiotics, which also includes all prescriptions made in primary care. We found a largely similar pattern for these outcomes, but while the absolute risk difference was larger for most comparisons due to overall higher incidence rates, the relative risk was smaller than for serious infections.

This study has certain limitations. This includes a lack of data on tobacco smoking status, but since Sweden has a very low rate of daily smokers (6% of the general population in 2021^28^) the impact is likely to be small. We also chose not to analyse cumulative dose or dosing intervals due to a significant degree of data missingness, though a large majority can be expected to have been exposed to bi-annual infusions with 500 mg of rituximab, at least until the COVID-19 pandemic outbreak. A further limitation is that as we did not have access to laboratory data, we could not include immunoglobulin levels or lymphocyte counts in the models to potentially identify relevant subgroups among rituximab-exposed individuals varying in infection risk.

In sum, we find an approximately doubled risk of serious infections with rituximab compared with other DMTs, regardless if used first line or as an escalation or switch agent, where the magnitude of risk increase appears to be stable over exposure time. This information is important for patients and treating neurologists in discussions of the benefit–risk balance with different treatment strategies. In parallel, additional efforts should be made to further develop risk mitigation strategies to diminish treatment-related risks with antiCD20 therapies.

## supplementary material

10.1136/jnnp-2023-333206 online supplemental file 1

## Data Availability

Release of data requires ethical approval for a specified research purpose.
