# Supplementary material for: Impact of previous treatment history and B-cell depletion treatment duration on infection risk in relapsing-remitting multiple sclerosis: a nationwide cohort study
Source: J Neurol Neurosurg Psychiatry. 2024 May 14;95(12):e333206. doi: 10.1136/jnnp-2023-333206 (PMC11671883; doi:10.1136/jnnp-2023-333206)
Supplement: online supplemental file 1 [file jnnp-95-12-s001.pdf]

Supplementary Online Content

Virtanen S, Piehl F, Frisell T

Impact of previous treatment history and B-cell depletion treatment duration on infection risk in relapsing-remitting multiple sclerosis: A nationwide cohort study

Contents

Table S1: Definition of outcomes 2
Table S2: Events, Incidence Rates, and Cox Proportional Hazard Models in the Full Cohort 3
Table S3: Baseline characteristics in the switch cohort 4
Figure S1: Drug survival – proportion of patients remaining on rituximab treatment over time 5
Table S4: Events, Incidence Rates, and Cox Proportional Hazard Models in the Full Cohort with follow up until February 29, 2020 6
Table S5: Events, Incidence Rates, and Cox Proportional Hazard Models in the Switch Cohort with follow up until February 29, 2020 7
Figure S2: Infection rate in rituximab by time on treatment, follow up until February 29, 2020 8
Table S6: Events, Incidence Rates, and Cox Proportional Hazard Models in the Switch Cohort where duration of previous treatment is ≥ 1 year 9

Table S1: Definition of outcomes

Outcome	Component	Source	Coding	Included codes
**Severe infections**	Hospitalization for infection	Main or contributory diagnosis in the inpatient component of Patient Register	ICD-10	A00-B99, D73.3, E06.0, E32.1, G00-G02, G04.2, G05-G07, H00.0, H44.0, H60.0-H60.3, H66-H67, H70, I30.1, I40.0, J00-J22, J32, J34.0, J36, J38.3, J39.0-J39.1, J44.0, J85, J86, K04.4, K04.6, K04.7, K10.2, K11.3, K12.2, K14.0, K57.0, K57.2, K57.4, K57.8, K61, K63.0, K65.0, K65.1, K65.2, K65.9, L00-L08, L30.3, M00-M01, M46.2-M46.5, M60.0, M65.0, M71.0, M71.1, M72.6, M86, N10, N11, N12, N13.6, N15.1, N15.9, N30.0 N30.8, N30.9, N34.0, N34.1, N34.2, N390, N41.2, N43.1, N45.2, N45.3, N45.4, N48.2, N61, N70, N73, N75.1, U07.1, U07.2
	Fatal infections	Causes of Death registry, underlying cause of death	ICD-10	As above
**Outpatient infections**	Specialist care outpatient infections	Main or contributory diagnosis in the outpatient component of Patient Register	ICD-10	As above
**Antibiotics**	Any systemic antibacterial	Prescribed drug registry	ATC	J01

Note: ICD-10 codes without a decimal place include all subdiagnoses

Table S2: Events, Incidence Rates, and Cox Proportional Hazard Models in the Full Cohort

	N	Events	PYR	IR	Crude HR (95% CI)	HR (95% CI)[1]	HR (95% CI)[2]	HR (95% CI)[3]
**Serious infection**								
**Rituximab first line**	1458	98	4308.4	22.7 (18.5-27.5)	Ref.	Ref.	Ref.	Ref.
**Rituximab later line**	3236	255	11918.2	21.4 (18.9-24.1)	0.94 (0.74-1.18)	0.94 (0.75-1.19)	0.87 (0.69-1.11)	0.82 (0.63-1.07)
**Other DMT first line**	2774	69	6663.3	10.4 (8.1-12.9)	0.46 (0.34-0.62)	0.47 (0.35-0.65)	0.45 (0.32-0.62)	0.48 (0.34-0.68)
**Other DMT later line**	3275	121	10640.4	11.4 (9.4-13.5)	0.50 (0.38-0.65)	0.50 (0.38-0.66)	0.49 (0.37-0.64)	0.48 (0.35-0.67)
**General population**	20308	521	79018.3	6.6 (6.0-7.2)	0.29 (0.23-0.36)	0.29 (0.23-0.36)	0.29 (0.23-0.36)	
**Outpatient infection**								
**Rituximab first line**	1458	228	4002.7	57.0 (49.8-64.6)	Ref.	Ref.	Ref.	Ref.
**Rituximab later line**	3236	581	11032.6	52.7 (48.5-57.0)	0.94 (0.81-1.10)	0.94 (0.81-1.10)	0.90 (0.77-1.06)	0.90 (0.75-1.08)
**Other DMT first line**	2774	230	6353.7	36.2 (31.7-41.0)	0.63 (0.53-0.76)	0.61 (0.51-0.74)	0.64 (0.52-0.77)	0.63 (0.52-0.78)
**Other DMT later line**	3275	371	10020.4	37.0 (33.4-40.9)	0.66 (0.56-0.78)	0.66 (0.56-0.78)	0.69 (0.57-0.82)	0.70 (0.57-0.85)
**General population**	20308	1705	75678.3	22.5 (21.5-23.6)	0.41 (0.35-0.47)	0.41 (0.35-0.47)	0.41 (0.36-0.47)	
**Systemic antibacterial**								
**Rituximab first line**	1458	583	2991.0	194.9 (179.4-211.1)	Ref.	Ref.	Ref.	Ref.
**Rituximab later line**	3236	1659	7520.5	220.6 (210.1-231.3)	1.17 (1.06-1.28)	1.10 (1.00-1.21)	1.04 (0.94-1.14)	1.03 (0.92-1.14)
**Other DMT first line**	2774	863	4905.1	175.9 (164.4-187.9)	0.90 (0.81-1.00)	0.81 (0.73-0.90)	0.79 (0.70-0.88)	0.79 (0.70-0.89)
**Other DMT later line**	3275	1359	7062.7	192.4 (182.3-202.8)	1.03 (0.94-1.14)	0.90 (0.82-1.00)	0.85 (0.77-0.95)	0.84 (0.75-0.95)
**General population**	20308	7341	57583.7	127.5 (124.6-130.4)	0.70 (0.64-0.76)	0.67 (0.61-0.73)	0.67 (0.62-0.73)	

Legend: N, number of patients; Events, number of patients with infection; PYR, person-years of follow-up; IR, incidence rate per 1000 PYR; HR, hazard ratio with 95% confidence interval from Cox proportional hazards model adjusting for:

1) Age, sex and start year
2) 1+ comorbidities and demographics
3) 2+ MS-specific

Table S3: Baseline characteristics in the switch cohort

	RTX with switch from NTZ	RTX with switch from INJ	RTX with switch from DMF	RTX with switch from FGL
**Number of observations**	886	786	626	346
**Start year, median (IQR)**	2017 (2015-2018)	2016 (2014-2018)	2018 (2016-2019)	2016 (2015-2018)
**Age in years, mean (SD)**	38.5 (10.4)	43.0 (9.8)	38.9 (10.5)	38.5 (9.7)
**Female, n (%)**	598 (67.5)	552 (70.2)	445 (71.1)	228 (65.9)
**Born in Sweden, n (%)**	765 (86.3)	666 (84.7)	541 (86.4)	295 (85.5)
**Education, 12+ yrs, n (%)**	393 (44.7)	376 (48.3)	299 (48.0)	155 (45.3)
**Years since MS diagnosis, mean (SD)**	7.7 (5.8)	7.7 (6.1)	5.6 (5.6)	8.0 (5.4)
**Hospitalized infection 5 yrs before DMT, n (%)**	35 (4.0)	22 (2.8)	25 (4.0)	14 (4.0)
**Infection outpatient care 5 yrs before DMT, n (%)**	120 (13.5)	93 (11.8)	103 (16.5)	61 (17.6)
**Antibiotics 5 yrs before DMT, n (%)**	511 (57.7)	420 (53.4)	368 (58.8)	221 (63.9)
**Charlson comorbidity index, mean (SD)**	0.1 (0.6)	0.1 (0.5)	0.1 (0.4)	0.1 (0.5)
**Any relapse year before DMT, n (%)**	95 (10.7)	183 (23.3)	143 (22.8)	88 (25.4)
**EDSS, mean (SD)**	2.0 (1.5)	1.7 (1.3)	1.6 (1.4)	1.9 (1.4)
**MSIS29 physical, mean (SD)**	1.8 (0.8)	1.7 (0.8)	1.7 (0.7)	1.9 (0.9)
**MSIS29 psychological, mean (SD)**	2.2 (0.9)	2.1 (0.9)	2.2 (0.9)	2.2 (1.0)
**Duration of previous DMT, years, mean (SD)**	3.5 (2.9)	5.5 (4.6)	1.9 (1.5)	2.5 (1.8)
**History of natalizumab, n (%)**	886 (100.0)	15 (1.9)	39 (6.2)	134 (38.7)
**History of fingolimod, n (%)**	99 (11.2)	5 (0.6)	28 (4.5)	346 (100.0)
**History of dimethyl fumarate, n (%)**	49 (5.5)	38 (4.8)	626 (100.0)	29 (8.4)
**1 previous treatment, n (%)**	266 (30.0%)	658 (83.7%)	312 (49.8%)	50 (14.5%)
**2 previous treatments, n (%)**	429 (48.4%)	72 (9.2%)	225 (35.9%)	158 (45.7%)
**3+ previous treatments, n (%)**	191 (21.6%)	56 (7.1%)	89 (14.2%)	138 (39.9%)
**Reason for previous treatment stop: Adverse events, n (%)**	45 (5.1%)	175 (22.3%)	245 (39.1%)	79 (22.8%)
**Reason for previous treatment stop: Lost of effect, n (%)**	76 (8.6%)	423 (53.8%)	294 (47.0%)	196 (56.6%)
**Reason for previous treatment stop: Other, n (%)**	765 (86.3%)	188 (23.9%)	87 (13.9%)	71 (20.5%)

Legend: RTX, rituximab; NTZ, natalizumab, INJ, injectables; DMF, dimethyl fumarate; FGL, fingolimod

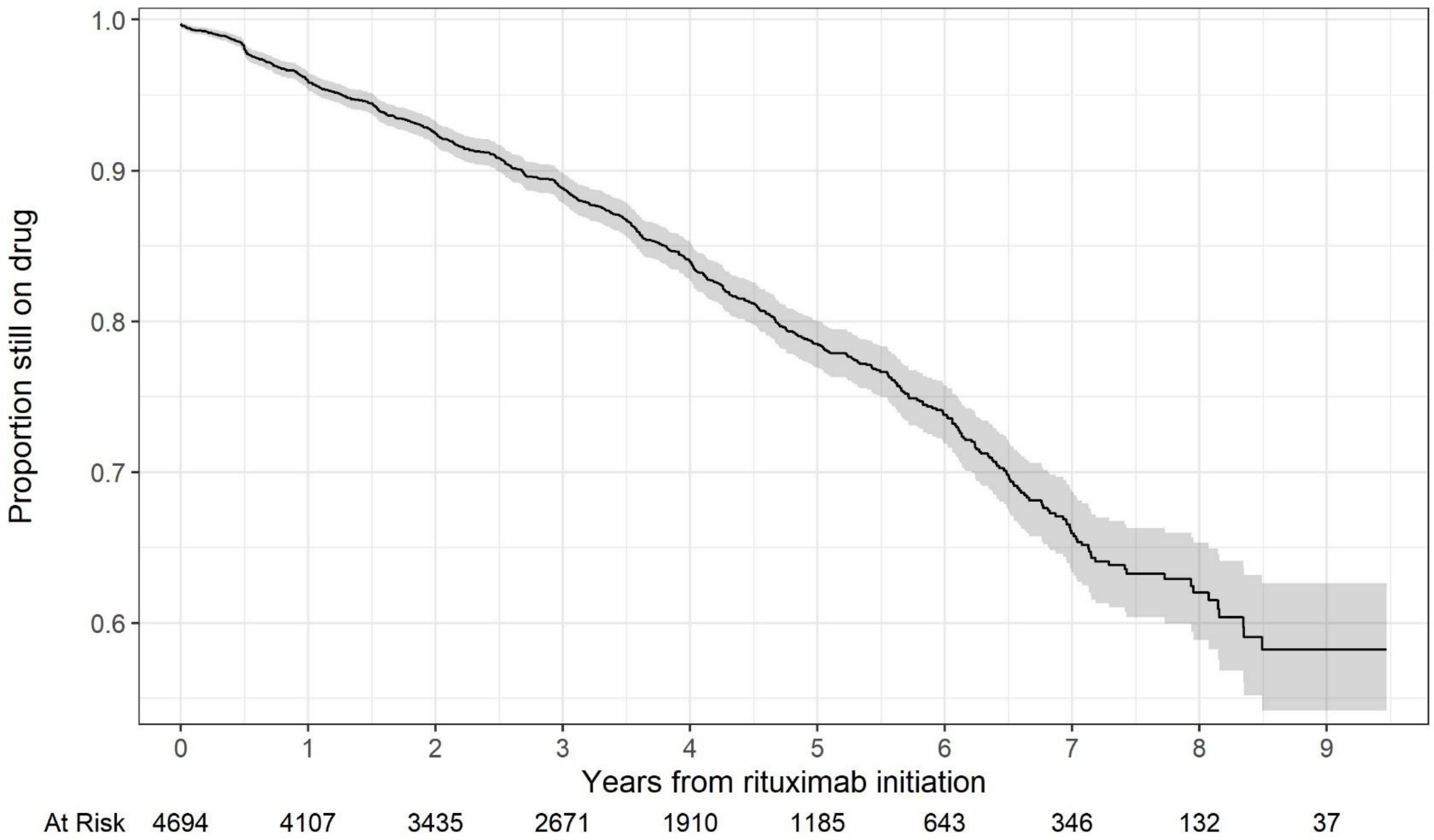

Figure S1: Drug survival – proportion of patients remaining on rituximab treatment over time

Table S4: Events, Incidence Rates, and Cox Proportional Hazard Models in the Full Cohort with follow up until February 29, 2020

	N	Events	PYR	IR	Crude HR (95% CI)	HR (95% CI)[1]	HR (95% CI)[2]	HR (95% CI)[3]
**Serious infection**								
**Rituximab first line**	1230	54	2795.3	19.3 (14.5-24.8)	Ref.	Ref.	Ref.	Ref.
**Rituximab later line**	2970	162	8670.1	18.7 (15.9-21.7)	0.97 (0.71-1.32)	0.96 (0.71-1.31)	0.89 (0.65-1.21)	0.85 (0.60-1.20)
**Other DMT first line**	2410	51	5526.7	9.2 (6.9-11.9)	0.48 (0.33-0.70)	0.44 (0.30-0.65)	0.42 (0.28-0.62)	0.44 (0.29-0.67)
**Other DMT later line**	3120	103	9088.3	11.3 (9.3-13.6)	0.59 (0.42-0.83)	0.55 (0.39-0.76)	0.53 (0.37-0.75)	0.51 (0.34-0.76)
**General population**	18100	344	54363.9	6.3 (5.7-7.0)	0.33 (0.25-0.44)	0.33 (0.24-0.44)	0.33 (0.25-0.44)	
**Outpatient infection**								
**Rituximab first line**	1230	152	2622.5	58.0 (49.1-67.5)	Ref.	Ref.	Ref.	Ref.
**Rituximab later line**	2970	428	8128.3	52.7 (47.8-57.8)	0.92 (0.77-1.11)	0.93 (0.77-1.12)	0.89 (0.74-1.08)	0.91 (0.74-1.13)
**Other DMT first line**	2410	195	5286.5	36.9 (31.9-42.2)	0.64 (0.52-0.79)	0.61 (0.49-0.76)	0.63 (0.51-0.79)	0.62 (0.49-0.79)
**Other DMT later line**	3120	329	8619.2	38.2 (34.2-42.4)	0.67 (0.55-0.81)	0.66 (0.54-0.81)	0.69 (0.56-0.85)	0.69 (0.54-0.87)
**General population**	18100	1195	52399.1	22.8 (21.5-24.1)	0.40 (0.34-0.48)	0.40 (0.34-0.48)	0.42 (0.35-0.49)	
**Systemic antibacterial**								
**Rituximab first line**	1230	449	1999.7	224.5 (204.2-245.8)	Ref.	Ref.	Ref.	Ref.
**Rituximab later line**	2970	1384	5739.3	241.1 (228.6-254.0)	1.10 (0.99-1.23)	1.06 (0.96-1.18)	1.00 (0.90-1.12)	0.97 (0.86-1.09)
**Other DMT first line**	2410	770	4152.3	185.4 (172.6-198.8)	0.84 (0.75-0.94)	0.79 (0.70-0.89)	0.77 (0.68-0.87)	0.75 (0.66-0.86)
**Other DMT later line**	3120	1270	6259.2	202.9 (191.9-214.2)	0.95 (0.85-1.06)	0.89 (0.80-0.99)	0.84 (0.75-0.94)	0.80 (0.70-0.91)
**General population**	18100	5798	41095.6	141.1 (137.5-144.7)	0.66 (0.60-0.73)	0.64 (0.58-0.71)	0.65 (0.59-0.71)	

Legend: N, number of patients; Events, number of patients with infection; PYR, person-years of follow-up; IR, incidence rate per 1000 PYR; HR, hazard ratio with 95% confidence interval from Cox proportional hazards model adjusting for:

1) Age, sex and start year
2) 1+ comorbidities and demographics
3) 2+ MS-specific

Table S5: Events, Incidence Rates, and Cox Proportional Hazard Models in the Switch Cohort with follow up until February 29, 2020

	N	Events	PYR	IR	Crude HR (95% CI)	HR (95% CI)[1]	HR (95% CI)[2]	HR (95% CI)[3]	HR (95% CI)[4]
**Serious infection**									
**RTX with switch from NTZ**	774	54	2387.4	22.6 (17.0-29.0)	Ref.	Ref.	Ref.	Ref.	Ref.
**RTX with switch from INJ**	755	38	2613.0	14.5 (10.3-19.5)	0.64 (0.42-0.97)	0.60 (0.39-0.93)	0.63 (0.41-0.98)	0.67 (0.42-1.07)	0.71 (0.42-1.19)
**RTX with switch from DMF**	564	21	1211.6	17.3 (10.7-25.5)	0.78 (0.47-1.30)	0.85 (0.50-1.44)	0.80 (0.48-1.36)	0.82 (0.47-1.42)	0.82 (0.47-1.42)
**RTX with switch from FGL**	336	20	1062.7	18.8 (11.5-27.9)	0.82 (0.49-1.37)	0.86 (0.51-1.44)	0.80 (0.48-1.34)	0.84 (0.49-1.44)	0.82 (0.48-1.40)
**Outpatient infection**									
**RTX with switch from NTZ**	774	131	2214.4	59.2 (49.5-69.7)	Ref.	Ref.	Ref.	Ref.	Ref.
**RTX with switch from INJ**	755	107	2482.2	43.1 (35.3-51.6)	0.73 (0.57-0.95)	0.75 (0.57-0.98)	0.76 (0.58-1.00)	0.80 (0.60-1.07)	0.85 (0.63-1.17)
**RTX with switch from DMF**	564	60	1142.5	52.5 (40.1-66.6)	0.89 (0.65-1.20)	0.83 (0.61-1.15)	0.79 (0.58-1.09)	0.77 (0.55-1.08)	0.78 (0.55-1.09)
**RTX with switch from FGL**	336	58	986.3	58.8 (44.7-74.9)	0.99 (0.73-1.34)	1.01 (0.74-1.37)	0.92 (0.67-1.26)	0.99 (0.71-1.36)	0.96 (0.69-1.33)
**Systemic antibacterial**									
**RTX with switch from NTZ**	774	385	1544.1	249.3 (225.1-274.9)	Ref.	Ref.	Ref.	Ref.	Ref.
**RTX with switch from INJ**	755	383	1714.6	223.4 (201.6-246.3)	0.91 (0.79-1.05)	0.87 (0.76-1.01)	0.91 (0.79-1.05)	0.91 (0.78-1.06)	0.95 (0.80-1.12)
**RTX with switch from DMF**	564	215	855.0	251.4 (219.0-286.2)	0.97 (0.82-1.14)	0.97 (0.81-1.15)	0.95 (0.80-1.14)	0.94 (0.78-1.14)	0.95 (0.79-1.14)
**RTX with switch from FGL**	336	162	670.6	241.6 (205.8-280.2)	0.97 (0.80-1.16)	0.98 (0.82-1.19)	0.94 (0.78-1.14)	0.96 (0.78-1.17)	0.94 (0.77-1.14)

Legend: RTX, rituximab; NTZ, natalizumab, INJ, injectables; DMF, dimethyl fumarate; FGL, fingolimod. N, number of patients; Events, number of patients with infection; PYR, person-years of follow-up; IR, incidence rate per 1000 PYR; HR, hazard ratio with 95% confidence interval from Cox proportional hazards model adjusting for:

1) Age, sex and start year
2) 1+ comorbidities and demographics
3) 2+ MS-specific
4) 3+ treatment history

Outcome	N	Events	PYR	IR	HR (95% CI)
**Serious infection**					
Year 1	4200	61	3779.8	16.1 (12.3-20.4)	Ref.
Year 2	3335	61	2908.2	21.0 (16.0-26.6)	1.28 (0.90-1.83)
Year 3	2474	37	2098.0	17.6 (12.4-23.8)	1.04 (0.68-1.59)
Year 4	1733	31	1350.5	23.0 (15.6-31.7)	1.35 (0.86-2.12)
Year 5	979	21	721.3	29.1 (18.0-42.8)	1.51 (0.87-2.60)
Year 6	531	3	390.4	7.7 (1.6-18.5)	0.30 (0.07-1.27)
Year 7	257	1	166.0	6.0 (0.2-22.2)	0.32 (0.04-2.39)
Year 8	100	1	50.4	19.9 (0.5-73.2)	1.27 (0.17-9.42)
Year 9	12	0	1.0	0.0	0.00 (0.00-0.00)
**Outpatient infection**					
Year 1	4200	216	3704.1	58.3 (50.8-66.3)	Ref.
Year 2	3207	153	2747.4	55.7 (47.2-64.9)	0.98 (0.79-1.21)
Year 3	2293	88	1922.2	45.8 (36.7-55.8)	0.80 (0.61-1.03)
Year 4	1564	64	1210.0	52.9 (40.7-66.6)	0.96 (0.71-1.29)
Year 5	868	32	636.2	50.3 (34.4-69.2)	0.93 (0.62-1.40)
Year 6	471	21	339.9	61.8 (38.2-90.9)	1.29 (0.78-2.11)
Year 7	219	4	145.3	27.5 (7.5-60.3)	0.54 (0.17-1.73)
Year 8	86	2	44.9	44.6 (5.4-124.1)	1.34 (0.32-5.53)
Year 9	10	0	0.8	0.0	0.00 (0.00-0.01)
**Systemic antibacterial**					
Year 1	4200	870	3357.0	259.2 (242.2-276.7)	Ref.
Year 2	2605	492	2035.2	241.7 (220.9-263.6)	0.93 (0.83-1.04)
Year 3	1547	275	1193.7	230.4 (203.9-258.4)	0.91 (0.79-1.05)
Year 4	883	112	644.7	173.7 (143.0-207.3)	0.69 (0.56-0.86)
Year 5	443	48	305.6	157.1 (115.8-204.5)	0.68 (0.51-0.92)
Year 6	209	26	137.2	189.5 (123.8-269.0)	0.80 (0.53-1.19)
Year 7	81	10	50.1	199.6 (95.7-341.0)	0.88 (0.46-1.71)
Year 8	27	0	15.3	0.0	0.00 (0.00-0.00)
Year 9	4	0	0.3	0.0	0.00 (0.00-0.00)

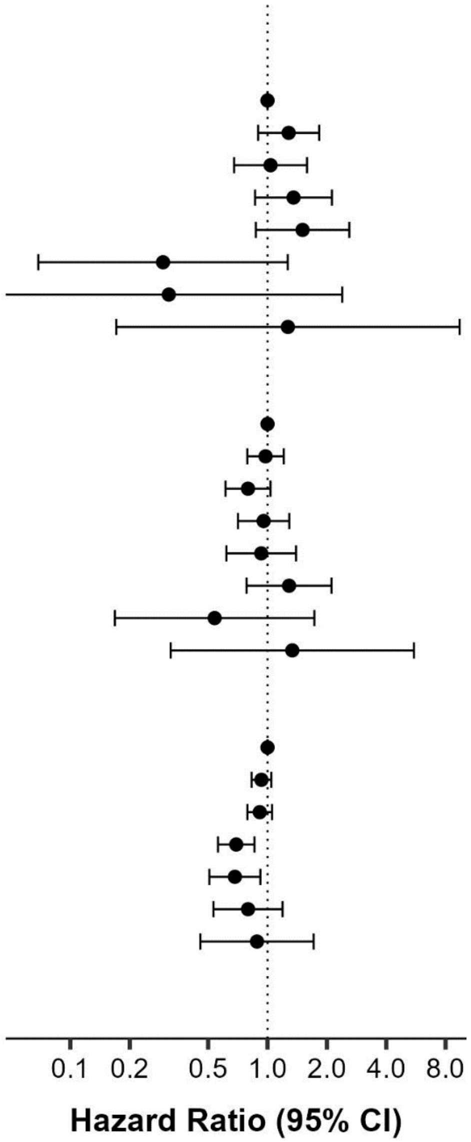

Figure S2: Infection rate in rituximab by time on treatment, follow up until February 29, 2020

Legend: N, number of patients; Events, number of patients with infection; PYR, person-years of follow-up; IR, incidence rate per 1000 PYR; HR, hazard ratio with 95% confidence interval from Cox proportional hazards model adjusting for age, sex, start year, comorbidities, demographics and MS clinical characteristics.

Table S6: Events, Incidence Rates, and Cox Proportional Hazard Models in the Switch Cohort where duration of previous treatment is ≥ 1 year

	N	Events	PYR	IR	Crude HR (95% CI)	HR (95% CI)[1]	HR (95% CI)[2]	HR (95% CI)[3]	HR (95% CI)[4]
**Serious infection**									
**RTX with switch from NTZ**	665	59	2646.1	22.3 (17.0-28.3)	Ref.	Ref.	Ref.	Ref.	Ref.
**RTX with switch from INJ**	665	41	2900.2	14.1 (10.1-18.8)	0.63 (0.42-0.94)	0.58 (0.39-0.88)	0.61 (0.40-0.93)	0.71 (0.46-1.10)	0.68 (0.42-1.09)
**RTX with switch from DMF**	398	25	1110.0	22.5 (14.6-32.2)	1.01 (0.63-1.61)	0.90 (0.56-1.45)	0.93 (0.58-1.49)	1.15 (0.70-1.89)	1.15 (0.70-1.90)
**RTX with switch from FGL**	258	22	1013.8	21.7 (13.6-31.7)	0.97 (0.59-1.58)	1.00 (0.61-1.63)	0.97 (0.59-1.60)	1.15 (0.69-1.93)	1.19 (0.70-1.99)
**Outpatient infection**									
**RTX with switch from NTZ**	665	135	2407.3	56.1 (47.0-65.9)	Ref.	Ref.	Ref.	Ref.	Ref.
**RTX with switch from INJ**	665	111	2712.7	40.9 (33.7-48.9)	0.74 (0.57-0.95)	0.76 (0.58-0.99)	0.78 (0.60-1.02)	0.86 (0.65-1.14)	0.88 (0.64-1.21)
**RTX with switch from DMF**	398	58	1052.7	55.1 (41.8-70.2)	0.94 (0.69-1.28)	0.90 (0.65-1.23)	0.94 (0.68-1.30)	0.97 (0.69-1.38)	0.97 (0.69-1.38)
**RTX with switch from FGL**	258	53	904.2	58.6 (43.9-75.4)	1.03 (0.75-1.41)	1.02 (0.74-1.40)	0.96 (0.69-1.34)	1.01 (0.71-1.43)	1.00 (0.70-1.41)
**Systemic antibacterial**									
**RTX with switch from NTZ**	665	354	1630.5	217.1 (195.1-240.3)	Ref.	Ref.	Ref.	Ref.	Ref.
**RTX with switch from INJ**	665	377	1788.2	210.8 (190.1-232.6)	0.97 (0.84-1.13)	0.92 (0.79-1.06)	0.96 (0.83-1.12)	0.98 (0.84-1.15)	1.01 (0.85-1.21)
**RTX with switch from DMF**	398	162	814.1	199.0 (169.5-230.8)	0.85 (0.71-1.03)	0.90 (0.74-1.09)	0.92 (0.75-1.12)	0.90 (0.73-1.12)	0.91 (0.73-1.12)
**RTX with switch from FGL**	258	124	637.0	194.6 (161.9-230.4)	0.88 (0.72-1.09)	0.90 (0.73-1.11)	0.87 (0.70-1.08)	0.93 (0.75-1.16)	0.92 (0.73-1.15)

Legend: RTX, rituximab; NTZ, natalizumab, INJ, injectables; DMF, dimethyl fumarate; FGL, fingolimod. N, number of patients; Events, number of patients with infection; PYR, person-years of follow-up; IR, incidence rate per 1000 PYR; HR, hazard ratio with 95% confidence interval from Cox proportional hazards model adjusting for:

1) Age, sex and start year
2) 1+ comorbidities and demographics
3) 2+ MS-specific
4) 3+ treatment history
